# Evaluating the potential for sperm DNA fragmentation testing to guide the use of ICSI for couples with non-severe male infertility

**DOI:** 10.1093/hropen/hoag011

**Published:** 2026-03-07

**Authors:** Yuanyuan Wang, Rui Wang, Ying Lian, Rui Yang, Jiangman Gao, Jianqiao Liu, Li Tang, Xiaoyan Liang, Yunxia Cao, Wen Li, Li Jin, Yimin Zhu, Junli Zhao, Guimin Hao, Huichun Wang, Ben W Mol, Rong Li, Jie Qiao

**Affiliations:** National Clinical Research Centre for Obstetrical and Gynaecological Diseases, State Key Laboratory of Female Fertility Promotion, Centre for Reproductive Medicine, Department of Obstetrics and Gynaecology, Peking University Third Hospital, Beijing, China; NHMRC Clinical Trials Centre, University of Sydney, Sydney, NSW, Australia; National Clinical Research Centre for Obstetrical and Gynaecological Diseases, State Key Laboratory of Female Fertility Promotion, Centre for Reproductive Medicine, Department of Obstetrics and Gynaecology, Peking University Third Hospital, Beijing, China; National Clinical Research Centre for Obstetrical and Gynaecological Diseases, State Key Laboratory of Female Fertility Promotion, Centre for Reproductive Medicine, Department of Obstetrics and Gynaecology, Peking University Third Hospital, Beijing, China; National Clinical Research Centre for Obstetrical and Gynaecological Diseases, State Key Laboratory of Female Fertility Promotion, Centre for Reproductive Medicine, Department of Obstetrics and Gynaecology, Peking University Third Hospital, Beijing, China; Centre for Reproductive Medicine, The Third Affiliated Hospital of Guangzhou Medical University, Guangzhou, China; Centre for Reproductive Medicine, First Affiliated Hospital of Kunming Medical University, Kunming, China; Centre for Reproductive Medicine, The Sixth Affiliated Hospital of Sun Yat-Sen University, Guangzhou, China; Centre for Reproductive Medicine, First Affiliated Hospital of Anhui Medical University, Hefei, China; The International Peace Maternity and Child Health Hospital, School of Medicine, Shanghai Jiao Tong University, Shanghai, China; Obstetrics and Gynaecology Hospital, Institute of Reproduction and Development, Fudan University, Shanghai, China; Women’s Hospital, Zhejiang University School of Medicine, Hangzhou, China; Centre for Reproductive Medicine, General Hospital of Ningxia Medical University, Yinchuan, China; Centre for Reproductive Medicine, The Second Hospital of Hebei Medical University, Shijiazhuang, China; Centre for Reproductive Medicine, Haidian Maternal and Child Health Hospital, Beijing, China; Department of Obstetrics and Gynaecology, School of Clinical Sciences, Monash University, Melbourne, VIC, Australia; National Clinical Research Centre for Obstetrical and Gynaecological Diseases, State Key Laboratory of Female Fertility Promotion, Centre for Reproductive Medicine, Department of Obstetrics and Gynaecology, Peking University Third Hospital, Beijing, China; National Clinical Research Centre for Obstetrical and Gynaecological Diseases, State Key Laboratory of Female Fertility Promotion, Centre for Reproductive Medicine, Department of Obstetrics and Gynaecology, Peking University Third Hospital, Beijing, China

**Keywords:** sperm DNA fragmentation, ICSI, IVF, assisted reproductive outcomes, non-severe male infertility

## Abstract

**STUDY QUESTION:**

In couples with non-severe male infertility, can sperm DNA fragmentation index (DFI) testing serve as a biomarker to identify couples who would benefit from ICSI over conventional IVF, based on cumulative live birth rate?

**SUMMARY ANSWER:**

Our secondary analysis of a randomized clinical trial (RCT) indicated that sperm DFI testing has limited value in identifying couples who would benefit from use of ICSI over IVF, based on cumulative live birth rate.

**WHAT IS KNOWN ALREADY:**

We recently demonstrated that in couples with non-severe male factor infertility, ICSI decreases cumulative live birth rates following the first transfer compared to IVF. However, it remains unclear whether sperm DFI testing can effectively guide treatment selection between ICSI and IVF.

**STUDY DESIGN, SIZE, DURATION:**

We used data of participants included in a randomized controlled trial comparing ICSI versus IVF in couples with non-severe male factor infertility. Participants had been recruited between April 2018 and November 2021 from seven centres in China, with follow-up outcomes collected as of 31 August 2023.

**PARTICIPANTS/MATERIALS, SETTING, METHODS:**

We included 953 of 2329 couples in whom the male partner had a baseline sperm DFI test from seven centres within the original RCT. The primary outcome was cumulative live birth, defined as a live birth following embryo transfers that occurred within 12 months after randomization within one oocyte retrieval cycle. The statistical analysis was performed based on an as-treated population, including 480 in the ICSI group and 473 in the IVF group. Multivariable fractional polynomial interaction analysis was performed to investigate non-linear interaction between DFI test and treatment effects of ICSI over IVF.

**MAIN RESULTS AND THE ROLE OF CHANCE:**

The median values of sperm DFI were 18.8% (interquartile range [IQR]: 12.2%∼26.3%) in the ICSI group, and 18.4% (IQR: 12.6%∼25.1%) in the IVF group. In each quantile of sperm DFI (Q1: DFI < 12.3%; Q2: 12.3≤DFI < 18.6%; Q3: 18.6≤DFI < 25.9%; Q4: DFI ≥ 25.9%), there were no significant differences of ICSI vs IVF on cumulative live birth (Q1: 36.7% vs 49.6%, adjusted odds ratio (aOR) = 0.61, 95% CI: 0.36 to 1.04; Q2: 51.8% vs 50.4%, aOR = 1.06, 95% CI: 0.60 to 1.84; Q3: 48.4% vs 56.3%, aOR = 0.78, 95% CI: 0.46 to 1.33; Q4: 45.2% vs 49.1%, aOR = 0.80, 95% CI: 0.47 to 1.37). Multivariable fractional polynomial interaction analysis showed no evidence of interaction between sperm DFI and the treatment effect of ICSI over IVF on cumulative live birth (*P* = 0.298).

**LIMITATIONS AND REASONS FOR CAUTION:**

First, sperm DFI testing was not routinely performed across all trial sites. Second, this study may be underpowered to detect differences in some outcomes, especially total fertilization failure, due to the very small number of events.

**WIDER IMPLICATIONS OF THE FINDINGS:**

Sperm DFI testing during the basal semen analysis has limited value in guiding the choice of fertilization methods for patients with non-severe male infertility. Based on the current evidence, sperm DFI testing should not be routinely recommended for this population.

**STUDY FUNDING/COMPETING INTEREST(S):**

This study was funded by the National Key Research and Development Program (2022YFC2703102 to Y.W.), and the Peking University Third Hospital (BYSYDL2022001 to J.Q., BYSYDL2024003 to Y.W., and BYSYZD2019007 to Y.L.). B.W.M. reports consultancy, travel support, and research funding from Merck KGaA and consultancy for Organon and Norgine.

**TRIAL REGISTRATION NUMBER:**

The original trial was registered at Clinicaltrials.gov: NCT03298633.

WHAT DOES THIS MEAN FOR PATIENTS? This study looked at whether the level of sperm DNA damage, measured by sperm DNA fragmentation index (DFI), can help couples with non-severe male infertility decide between ICSI and conventional IVF to improve their chances of having a baby.

Sperm DFI testing is sometimes used in clinical practice to help diagnose male infertility and guide the choice of fertilization method. However, there is ongoing debate about its usefulness, as previous studies have reported conflicting results.

In this study, nearly 1000 couples from a large trial in China were included. The results showed that the level of sperm DNA damage did not make much difference in live birth rates between ICSI and IVF. It was concluded that sperm DFI testing cannot be considered as a routine check for couples with non-severe male infertility to decide on fertilization methods.

## Introduction

Male factor infertility contributes to approximately 30% of infertility cases (Agarwal *et al.*, 2021; Centers for Disease Control and Prevention, 2022). Semen analysis, which includes the assessment of sperm concentration, motility, and morphology, is a crucial component of the initial fertility work-up for couples with infertility (World Health Organization, 2010). Controversy surrounds the assessment of sperm DNA fragmentation. This test was introduced in the 1980s, using the Sperm Chromatin Structure Assay (SCSA) (Evenson *et al.*, 1980). Since then, several different methods have been developed, including the TUNEL assay, the Comet assay, and the sperm chromatin dispersion (SCD) test. Over the past decades, SCSA and TUNEL assays have been more widely utilized compared with other methods (Baskaran *et al.*, 2019). These sperm DNA fragmentation index (DFI) tests have been considered as an addition to the diagnosis of male factor infertility and the choice between ICSI or conventional IVF as a method of insemination (Agarwal *et al.*, 2020; Ribas-Maynou *et al.*, 2021).

However, the value of sperm DFI testing remains a subject of debate. Inconsistent conclusions from different meta-analyses challenge its predictive value regarding assisted reproductive outcomes. Some meta-analyses indicated that a higher sperm DFI among patients undergoing IVF and/or ICSI was associated with higher miscarriage rates (Robinson *et al.*, 2012), lower pregnancy rates (Zhang *et al.*, 2015), and lower live birth rates (Osman *et al.*, 2015). Conversely, other meta-analyses found no significant differences in live birth rates or other pregnancy outcomes (such as implantation, pregnancy, and ongoing pregnancy) among patients receiving either IVF or ICSI (Cissen *et al.*, 2016; Ribas-Maynou *et al.*, 2021). Given the insufficient evidence for the relevance of sperm DFI tests in predicting pregnancy or guiding treatment decisions, the latest ESHRE Good Practice Recommendations on add-ons (Lundin *et al.*, 2023) suggests that sperm DFI testing is currently not recommended for routine clinical use. There is a strong recommendation from ESHRE calling for research in this area given the lack of prospective studies.

We recently showed in a large randomized clinical trial (RCT) that in couples with non-severe male factor infertility, ICSI does not increase live birth rate following the first transfer compared to IVF and even decreased cumulative live birth rates (Wang *et al.*, 2024). The findings are consistent with other recently published large trials comparing ICSI and IVF (Dang *et al.*, 2021; Berntsen *et al.*, 2025). A substantial proportion of the male partners in this trial underwent sperm DFI testing during the trial, yet it remains unclear whether this testing can effectively identify couples who would benefit from ICSI over IVF and therefore guide treatment selection. Therefore, we conducted a secondary analysis to assess whether sperm DFI testing can serve as a biomarker to identify couples who would benefit from ICSI compared to IVF.

## Materials and methods

### Study design and study population

This secondary analysis utilized a nested prospective cohort design within a multicentre, open-label, randomized controlled trial comparing ICSI versus IVF versus in couples with non-severe male infertility (Wang *et al.*, 2024). The participants of the original trial were recruited between April 2018 and November 2021 in 10 reproductive medicine centres across China. Detailed descriptions of the study protocol, data collections, and the results of the original study were published previously (Zheng *et al.*, 2019; Wang *et al.*, 2024). In 7 of the 10 centres, the sperm DFI test was done routinely or in a large percentage of patients. Patients from the other three centres where no or a few participants were tested for DFI were excluded. In the current analysis, we included participants who had a baseline sperm DFI test in one of the centres where sperm DFI testing was done frequently.

### Semen analysis and DFI testing

Semen samples during the initial evaluation were collected after between 2 and 7 days of abstinence. Semen analyses (basal semen testing) were performed by using CASA prior to randomization. All semen samples were prepared by discontinuous density gradient centrifugation or swim-up protocol according to local routines in each centre. Microscopy (200–400 times) was used to identify the presentation of serious abnormalities in sperm morphology. Sperm DFI was tested by using SCSA kits (CellPro Biotech Co., Ltd. Ningbo, China), which is a commonly used method in China and was accessible at all centres participating in this study. Standardized staff training and quality control across all labs of recruiting centres for the examination and processing of human semen were performed according to the fifth edition of the WHO laboratory manual (World Health Organization, 2010).

### Outcomes

In the current study, the primary outcome was cumulative live birth, defined as a live birth following transfers within 12 months after randomization of embryos created in a single IVF/ICSI cycle. The secondary outcomes included live birth after the first embryo transfer, ongoing pregnancy, clinical pregnancy, and total fertilization failure. The detailed definitions of outcomes were provided in the original study published previously (Zheng *et al.*, 2019; Wang *et al.*, 2024).

### Statistical analysis

The statistical analysis was performed based on an as-treated population that considered the treatment (ICSI or IVF) actually received by the participant, without regard to adherence to their randomization assignment (Smith *et al.*, 2021). Only couples with available data of sperm DFI were eligible for the current analysis. Baseline characteristics were described by using medians with interquartile ranges (IQRs), or counts with percentages where appropriate. Comparisons of baseline characteristics between groups (IVF vs ICSI) were done by using the Wilcoxon rank-sum test for continuous variables or using the Chi-square test for categorical variables. Baseline characteristics between participants included in this secondary analysis and those excluded were also compared using the same method. Histograms were used to understand the frequency distribution of DFI in each group.

To evaluate the treatment effects of ICSI vs IVF on each outcome at different DFI levels, we first divided the cohort into four quartiles (at 25th, 50th, and 75th centiles), and in each quartile, we performed logistic regression models with adjustment of centre. We calculated the adjusted odd ratios (aORs) with 95% CIs of ICSI vs IVF on each outcome in each quartile of DFI. Second, we considered DFI as a continuous variable and performed multivariable fractional polynomial interaction analyses in logistic regression models with adjustment of centre, in order to evaluate the non-linear association between sperm DFI value and the treatment effect of ICSI vs IVF on each outcome. According to the frequency distribution of DFI, we truncated sperm DFI values at the 0.5th and 99.5th percentiles to avoid the impact of outliers in the models. We restricted the analyses to linear (fp0), one-term fractional polynomials (fp1) and two-term fractional polynomials (fp2) for both main effects and interaction. The final model was determined according to the principle of minimized Akaike information criterion (Royston and Sauerbrei, 2004, 2009). Further details of the methods can be found in other examples where we have applied this method in the past (Hu *et al.*, 2022; Hunt *et al.*, 2024). Finally, we visualized the aORs with 95% CIs of treatment effects between groups at different baseline sperm DFI values. To facilitate clinical interpretation, we also produced a figure to illustrate the predicted absolute risks (probabilities with 95% CIs) in each group (ICSI and IVF) at different baseline sperm DFI values.

In post-hoc analyses of this study, to further evaluate the effects of sperm DFI on embryological outcomes, we performed linear regression models with adjustment of centre in each quartile of DFI and calculated the adjusted *P* values of ICSI vs IVF on numbers of available embryos and good quality embryos on Day 3. In each quartile of DFI, we also described the status of fresh/frozen-thawed or cleavage-stage/blastocyst embryo transfer in the first cycle in each group and thereby compared the differences between groups by using logistic regression models with adjustment of centre.

All statistical analyses were conducted by using SPSS version 23.0 (IBM, Armonk, NY, USA) and Stata version 18.0 (StataCorp LLC, College Station, TX, USA). A two-tailed *P* value < 0.05 was considered to indicate statistical significance.

## Results

**Figure 1.**
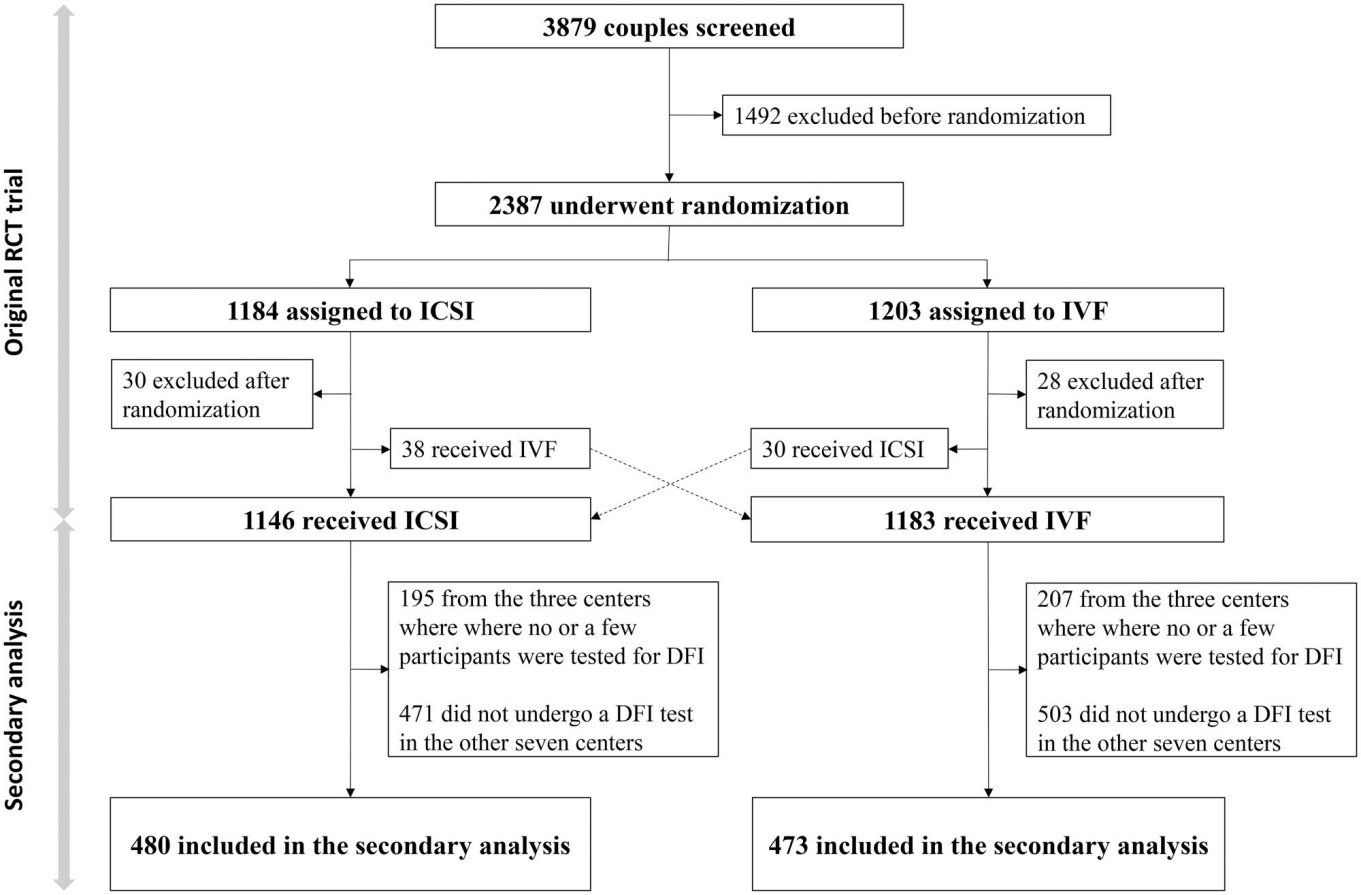
**Flowchart of study population.** DFI: DNA fragmentation index.

Out of the original 2387 couples randomized in the trial, 58 couples withdrew after randomization, and a total of 2329 couples received either ICSI (1146) or IVF (1183). Finally, 953 couples (480 in the ICSI group and 473 in the IVF group) from seven centres were finally included in the secondary analysis (Fig. 1). The comparison of baseline characteristic between included couples and excluded couples in each group can been seen in Supplementary Tables S1 and S2.

**Figure 2.**
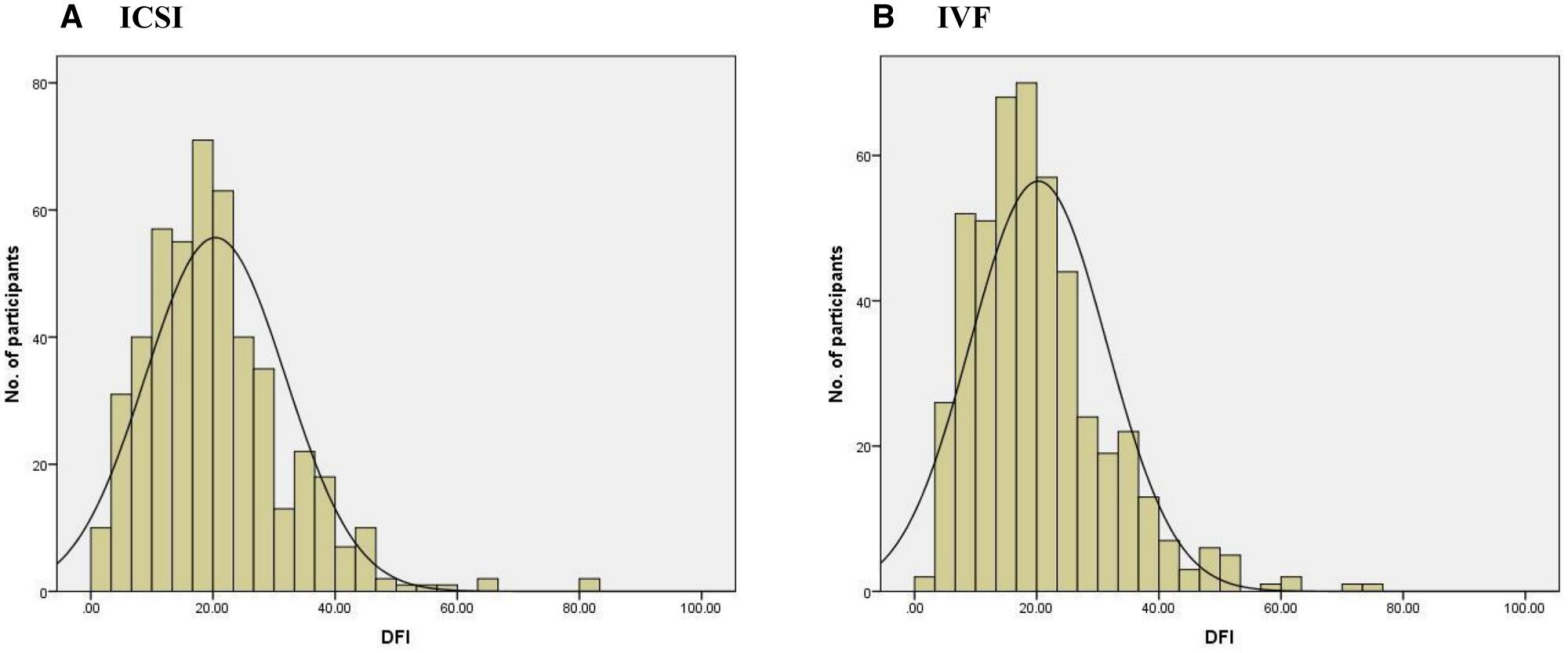
**The distribution of DFI in the study population.** DFI: DNA fragmentation index.

In the couples included in the sperm DFI analysis, the baseline characteristics between the two groups were overall comparable (Supplementary Table S3). The median values of sperm DFI were 18.8% (IQR: 12.2%∼26.3%) in the ICSI group, and 18.4% (IQR: 12.6%∼25.1%) in the IVF group, with no significant difference between the two groups (*P* = 0.945) (Supplementary Table S3, Fig. 2).

In each quartile of sperm DFI (Q1: DFI < 12.3%; Q2: 12.3≤DFI < 18.6%; Q3: 18.6≤DFI < 25.9%; Q4: DFI ≥ 25.9%), there were no significant differences of ICSI vs IVF on cumulative live birth, adjusting for centre (Q1: 36.7% vs 49.6%, aOR = 0.61, 95% CI: 0.36 to 1.04; Q2: 51.8% vs 50.4%, aOR = 1.06, 95% CI: 0.60 to 1.84; Q3: 48.4% vs 56.3%, aOR = 0.78, 95% CI: 0.46 to 1.33; Q4: 45.2% vs 49.1%, aOR = 0.80, 95% CI: 0.47 to 1.37). Importantly, there was no subgroup where ICSI was better than IVF. No between-group differences within each quantile were observed on live birth, ongoing pregnancy, or clinical pregnancy. No between-group significant differences on total fertilization failure were observed in Q1, Q2, and Q3, while the ICSI group in Q4 had a lower risk of total fertilization failure compared with the IVF group (1.6% vs 7.0%, aOR = 0.18, 95% CI: 0.04 to 0.88). The detailed results are shown in Table 1.

**Table 1.** Treatment effects on pregnancy outcomes in each quartile of sperm DFI.

	ICSI n/N (%)	IVF n/N (%)	**aOR (95% CI)** *	Forest plot
**Cumulative live birth**				
Q1 (DFI < 12.3%)	44/120 (36.7)	58/117 (49.6)	0.61 (0.36, 1.04)	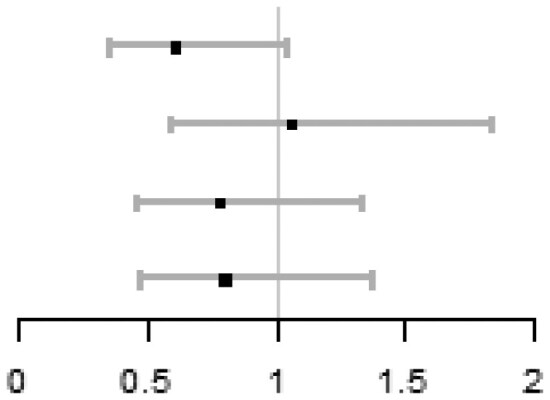
Q2 (12.3 ≤ DFI < 18.6%)	59/114 (51.8)	62/123 (50.4)	1.06 (0.60, 1.84)	
Q3 (18.6 ≤ DFI < 25.9%)	59/122 (48.4)	67/119 (56.3)	0.78 (0.46, 1.33)	
Q4 (DFI ≥ 25.9%)	56/124 (45.2)	56/114 (49.1)	0.80 (0.47, 1.37)	
**Live birth**				
Q1 (DFI < 12.3%)	33/120 (27.5)	38/117 (32.5)	0.87 (0.49, 1.55)	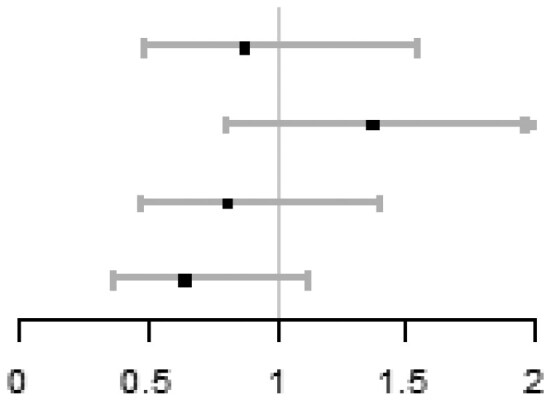
Q2 (12.3 ≤ DFI < 18.6%)	50/114 (43.9)	45/123 (36.6)	1.37 (0.80, 2.38)	
Q3 (18.6 ≤ DFI < 25.9%)	41/122 (33.6)	47/119 (39.5)	0.81 (0.47, 1.40)	
Q4 (DFI ≥ 25.9%)	39/124 (31.5)	47/114 (41.2)	0.64 (0.37, 1.12)	
**Ongoing pregnancy**				
Q1 (DFI < 12.3%)	33/120 (27.5)	38/117 (32.5)	0.87 (0.49, 1.55)	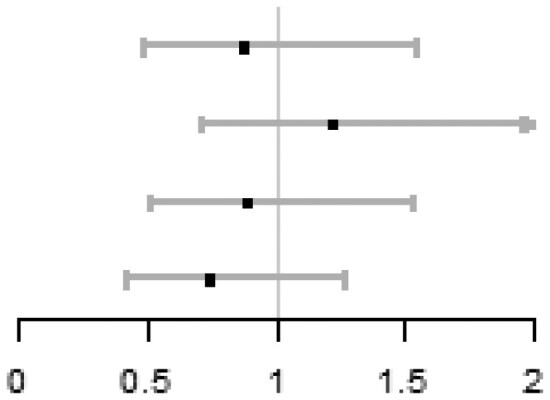
Q2 (12.3 ≤ DFI < 18.6%)	51/114 (44.7)	49/123 (39.8)	1.22 (0.71, 2.10)	
Q3 (18.6 ≤ DFI < 25.9%)	43/122 (35.2)	47/119 (39.5)	0.89 (0.52, 1.53)	
Q4 (DFI ≥ 25.9%)	43/124 (34.7)	47/114 (41.2)	0.74 (0.42, 1.27)	
**Clinical pregnancy**				
Q1 (DFI < 12.3%)	35/120 (29.2)	45/117 (38.5)	0.73 (0.42, 1.27)	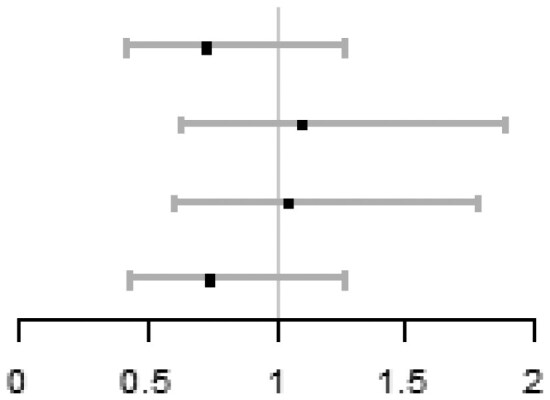
Q2 (12.3 ≤ DFI < 18.6%)	59/114 (51.8)	61/123 (49.6)	1.10 (0.64, 1.89)	
Q3 (18.6 ≤ DFI < 25.9%)	53/122 (43.4)	53/119 (44.5)	1.04 (0.61, 1.78)	
Q4 (DFI ≥ 25.9%)	49/124 (39.5)	52/114 (45.6)	0.74 (0.43, 1.27)	
**Total fertilization failure**				
Q1 (DFI < 12.3%)	9/120 (7.5)	4/117 (3.4)	2.50 (0.71, 8.78)	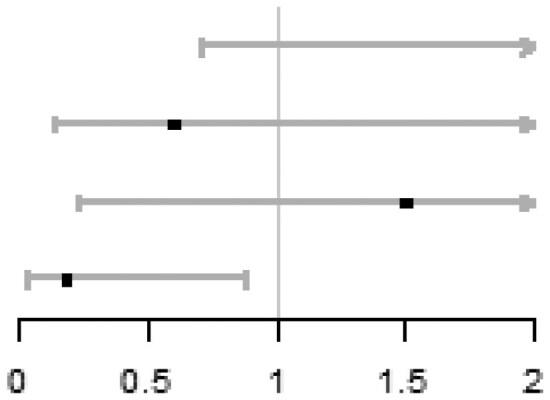
Q2 (12.3 ≤ DFI < 18.6%)	3/114 (2.6)	5/123 (4.1)	0.60 (0.14, 2.64)	
Q3 (18.6 ≤ DFI < 25.9%)	3/122 (2.5)	2/119 (1.7)	1.50 (0.24, 9.53)	
Q4 (DFI ≥ 25.9%)	2/124 (1.6)	8/114 (7.0)	0.18 (0.04, 0.88)	

* aOR (95% CI) was calculated by using logistic model with the adjustment of centre.

DFI: DNA fragmentation index; aOR: adjusted odds ratio.

**Figure 3.**
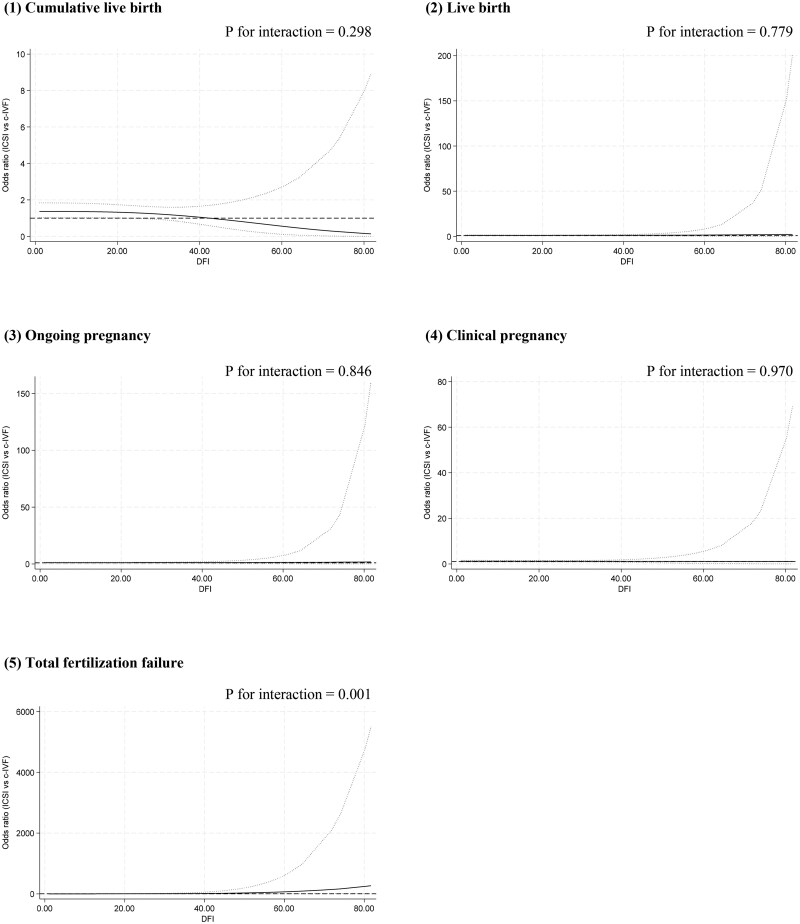
**The relative treatment effects of ICSI vs IVF at different DFI levels on outcomes.** Outcomes include (1) cumulative live birth, (2) live birth, (3) ongoing pregnancy, (4) clinical pregnancy, and (5) total fertilization failure. Multivariable fractional polynomial interaction analysis was performed; dotted lines: 95% CIs. DFI: DNA fragmentation index.

**Figure 4.**
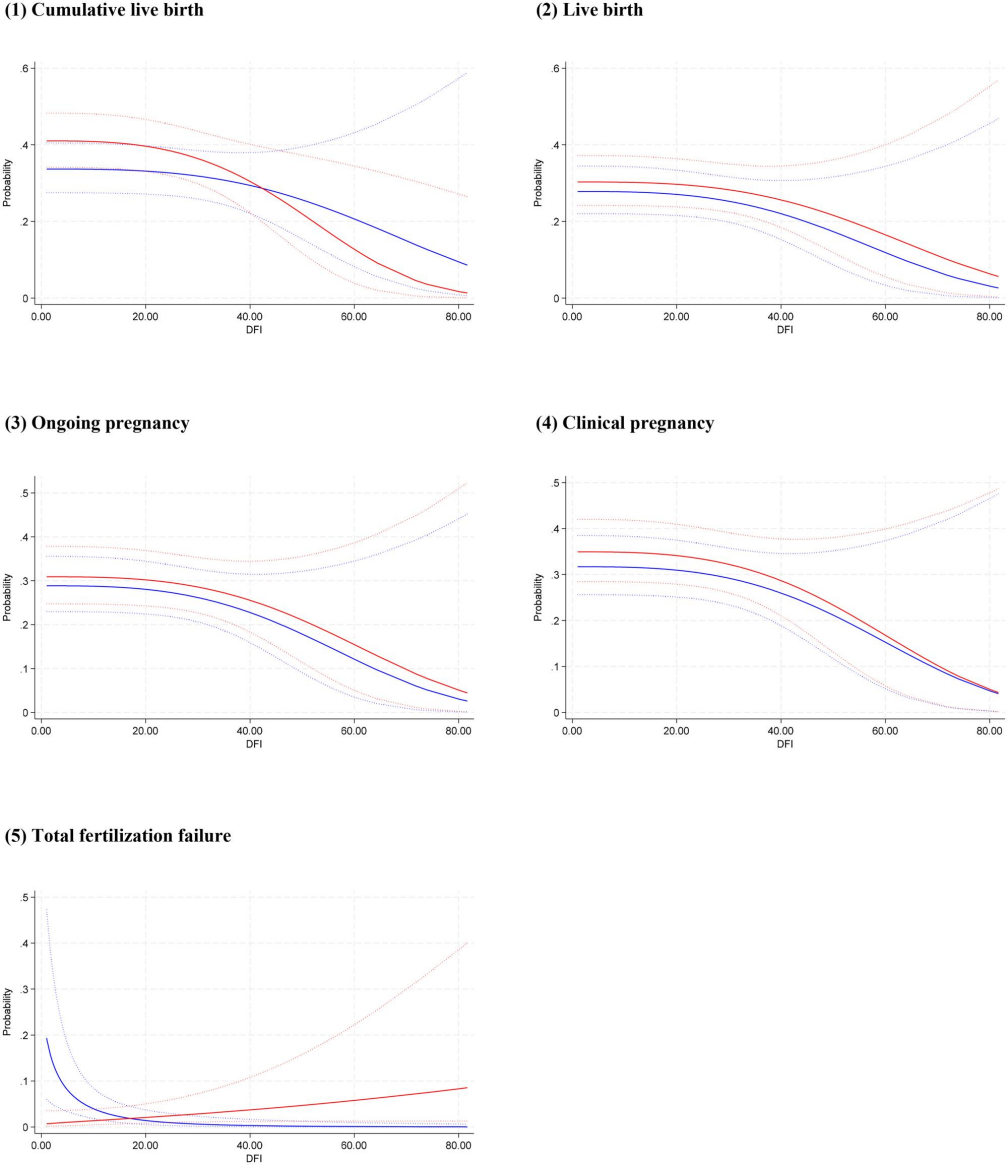
**Predicted outcomes in each group at different DFI levels.** Outcomes include (1) cumulative live birth, (2) live birth, (3) ongoing pregnancy, (4) clinical pregnancy, and (5) total fertilization failure. Multivariable fractional polynomial interaction analysis was performed; red line: ICSI, blue line: IVF, dotted lines: 95% CIs. DFI: DNA fragmentation index.

In the multivariable fractional polynomial interaction analyses, based on the principle of minimized Akaike information criterion, fp1 interaction models (power = 1) with the adjustment of centre were finally used for all outcomes in this study. Figure 3 shows treatment effect of ICSI vs IVF on each outcome at different sperm DFI levels, indicating no evidence of interaction between sperm DFI and the treatment effect on cumulative live birth (*P* = 0.298), live birth (*P* = 0.779), ongoing pregnancy (*P* = 0.846) and clinical pregnancy (*P* = 0.970), but a significant interaction on total fertilization failure (*P* = 0.001). Figure 4 shows that the predicted outcomes for the two groups persistently overlapped at different sperm DFI levels, indicating that the treatment effect of ICSI vs IVF on each outcome varies little across different sperm DFI levels.

In post-hoc analyses (Supplementary Table S4), in each quartile of sperm DFI, there were no significant differences between ICSI and IVF on number of available embryos or number of good quality embryos on Day 3 (all adjusted *P* values > 0.05). Embryo transfers in the first cycle (fresh or frozen-thawed; cleavage-stage or blastocyst) were generally comparable between the two groups (Supplementary Table S4).

## Discussion

In this secondary analysis of a multicentre randomized controlled trial involving couples with non-severe male factor infertility, we found no significant interaction between sperm DFI and the treatment effect of ICSI vs IVF on cumulative live birth, live birth, ongoing pregnancy, or clinical pregnancy. Therefore, this study does not support the use of sperm DFI to select candidate couples who would benefit from ICSI over IVF, based on fertility outcomes. Furthermore, subgroup analyses across different DFI levels showed no significant differences between ICSI and IVF in the number of available embryos or high-quality embryos on Day 3. This further reinforces the conclusion that sperm DFI testing should not be considered a routine test for couples with non-severe male infertility when deciding on fertilization methods.

Over the past two decades, there have been an increasing number of research publications on sperm DFI related to assisted reproductive outcomes (Baskaran *et al*., 2019). In the latest sixth edition of the WHO laboratory manual for the examination and processing of human semen, WHO has recommended the sperm DFI testing as part of the extended semen examination, but failed to provide recommendations on the specific medical indicators of its use, gold standard testing methods, or the diagnostic cut-off values, due to the lack of high-quality evidence (World Health Organization, 2021). Previous studies have reported that elevated sperm DFI may be associated with lower rates of clinical pregnancy and live birth; however, the quality of evidence is largely limited by the nature of retrospective design, significant heterogeneity and inconsistent conclusions across different studies (Robinson *et al.*, 2012; Cissen *et al.*, 2016; Ribas-Maynou *et al.*, 2021). The lack of high-quality evidence has led to inconsistent recommendations in clinical guidelines and the overuse of sperm DFI testing in clinical practice. For couples planning ART treatment, sperm DFI testing is recommended by the European Academy of Andrology (EAA) (Colpi *et al.*, 2018); recommended only for the cases with recurrent pregnancy loss or unexplained male infertility by the European Association of Urology (EAU) (Tharakan *et al.*, 2022); recommended only for the cases with recurrent pregnancy loss by ESHRE (ESHRE Guideline Group on RPL *et al.*, 2023; ESHRE Guideline Group on Unexplained Infertility, 2023), and the American Urological Association/American Society for Reproductive Medicine (AUA/ASRM) (Schlegel *et al.*, 2021a,b); while strongly not recommended by the Guideline of the German Society of Gynecology and Obstetrics, the Austrian Society of Gynecology and Obstetrics, and the Swiss Society of Gynecology and Obstetrics (DGGG, OEGGG, and SGGG) (Toth *et al.*, 2019). In many other countries including China, there have been no guidelines or consensus on the indications for sperm DFI testing. A global survey of experts on indications for sperm DFI testing in male infertility showed no consensus of opinions from 436 experts from 55 countries, 23.9% would assess sperm DFI for all cases with male infertility before ART (Farkouh *et al.*, 2023).

The findings of our study indicate that the treatment effects of ICSI vs IVF vary little across different sperm DFI levels in patients with non-severe male infertility, suggesting that the sperm DFI testing has limited value in guiding the use of ICSI or IVF. However, since the current methods of sperm DFI testing render the tested sperm unusable for ICSI, the sperm testing in our study was conducted during the basal semen analysis prior to ICSI/IVF. Recent evidence also showed limited value of sperm morphology in guiding treatment choice of ICSI and IVF in couples where the male partner had normal total sperm count and motility (Pham *et al.*, 2025). There is a need for better sperm testing techniques for the diagnosis of male infertility and subsequent treatment selection of ICSI or IVF. Currently, based on the available evidence, sperm DFI testing should not be recommended for routine use in this population. Since severe male factor contributes to less than 10% of infertility cases (Cooper *et al.*, 2010; Agarwal *et al.*, 2021; Mazzilli *et al.*, 2022), eliminating the overuse of sperm DFI testing in ART treatment is necessary.

From the results of this study, the ICSI group in Q4 (DFI ≥ 25.9%) had a lower rate of total fertilization failure compared with the IVF group. Although this may be a chance finding, detecting differences in total fertilization failure due to the very small number of events, we cannot rule out the possibility that ICSI may have a protective effect on fertilization in men with relatively higher sperm DFI. This is consistent with previous research findings (Bungum *et al.*, 2004; Tang *et al.*, 2021), as ICSI was originally developed to overcome fertilization failure caused by severe male infertility (including higher sperm DNA damage level). Therefore, for couples with non-severe male infertility, if total fertilization failure occurred in a previous IVF cycle, sperm DFI testing could be considered as a recommendation. If the DFI is ≥25, ICSI may be considered to avoid repeated fertilization failure. However, this recommendation requires further validation through well-designed prospective cohort studies or RCTs. Moreover, all semen samples in our study were prepared by discontinuous density gradient centrifugation or swim-up protocol according to the local routines in each centre. Although some studies showed that different sperm preparation methods might affect sperm DNA integrity (Raad *et al.*, 2021; Wen *et al.*, 2025), other studies showed no significant differences in fertility and pregnancy outcomes among such different methods (Dai *et al.*, 2020; Vagios *et al.*, 2023). Since the impact of different semen processing methods on the relationship between DFI and fertility outcomes remains unclear, we broadly adjusted for centre effects without further differentiating the effects of specific semen preparation techniques on the outcomes.

The strengths of this study include its nested prospective cohort design within a multicentre randomized controlled trial, the comparability of baseline characteristics between groups, and a relatively small proportion of loss to follow-up. However, this study also has several limitations. First, sperm DFI testing was not routinely performed across all recruiting sites, breaking the randomness of the study population in the original trial. Therefore, this secondary analysis is essentially a prospective cohort design rather than a randomized controlled trial. To control for this bias in the study population, we adjusted for centre effects in all logistic regression models, which also helps to address the inconsistencies in treatment protocols across different centres (e.g. ovulation induction protocols, endometrial preparation, etc.). Second, considering the standardization and accessibility of sperm DFI testing methods, we selected only SCSA assays for this study. Previous research indicates that sperm DFI testing method preferences vary by country, with SCSA and TUNEL being the most commonly used (Baskaran *et al.*, 2019). Although previous research has showed that SCSA and TUNEL are well correlated, and SCSA is more sensitive than TUNEL (Evenson *et al.*, 2016; Behdarvandian *et al.*, 2023), results obtained from different testing methods are not directly comparable and there is currently no universally accepted cutoff value for any specific testing method. Therefore, in this study, we treated sperm DFI as a continuous variable to evaluate treatment effect at each DFI value and also conducted subgroup analyses based on its quartile distribution to minimize the impact of testing methods on the interpretation of results. Besides the use of DFI threshold values, the method of sperm preparation, timing of measurement, and inter-laboratory variability across centres should be also acknowledged in the interpretation of the findings from this study. Third, there is currently no unified definition for non-severe male infertility, and the diagnostic criteria used vary slightly across different studies (Agarwal *et al.*, 2021; Berntsen *et al.*, 2025). In our study, we assessed this condition using two sperm parameters, including semen concentration (5–15 × 10^6^ sperm per ml) and progressive motility (10–32%) (Wang *et al.*, 2024). Additionally, nearly 80% of the participants had asthenozoospermia only (Wang *et al.*, 2024), which aligns with previous reports on characteristics of a similar population (Punab *et al.*, 2017; Wu *et al.*, 2021). However, our findings should be interpreted with caution outside of the trial population. Furthermore, the lack of several key outcomes (e.g. blastulation) and variables (e.g. endometrial preparation) precludes us from controlling these confounding factors or performing subgroup analyses. Therefore, future studies should account for the impacts of these confounding factors to comprehensively elucidate the effect of DFI on IVF/ICSI outcomes.

In conclusion, we found that the sperm DFI has limited value in guiding the choice of fertilization methods for patients with non-severe male infertility. Based on the current evidence, sperm DFI testing should not be routinely recommended for this population.

## Supplementary Material

hoag011_Supplementary_Data

## Data Availability

The data underlying this article will be shared upon reasonable request to the leading primary investigator of the study (jie.qiao@263.net).
