# Supplementary material for: Evaluating the potential for sperm DNA fragmentation testing to guide the use of ICSI for couples with non-severe male infertility
Source: Hum Reprod Open. 2026 Mar 7;2026(2):hoag011. doi: 10.1093/hropen/hoag011 (PMC12981913; doi:10.1093/hropen/hoag011)
Supplement: hoag011_Supplementary_Data [file hoag011_supplementary_data.zip › Supplementary-Table-S3-post adjudication clean.docx]

**Supplementary Table S3: Baseline** **characteristics of the study population included in the secondary analysis**

| **Characteristic** | **ICSI**  **(N=480)** | **IVF**  **(N=473)** | **Z/ꭓ^2^** | **P** |
| --- | --- | --- | --- | --- |
| Centre |  |  |  |  |
| 1 | 138 (28.7%) | 128 (27.1%) | 2.225 | 0.898 |
| 2 | 53 (11.0%) | 45 (9.5%) |  |  |
| 3 | 35 (7.3%) | 40 (8.5%) |  |  |
| 4 | 54 (11.3%) | 62 (13.1%) |  |  |
| 5 | 72 (15.0%) | 68 (14.4%) |  |  |
| 7 | 52 (10.8%) | 49 (10.4%) |  |  |
| 9 | 76 (15.8%) | 81 (17.1%) |  |  |
| Age (years) |  |  |  |  |
| Female | 34 (31, 38) | 34 (30, 38) | 0.448 | 0.654 |
| Male | 35 (31, 39) | 34 (31, 39) | 1.263 | 0.206 |
| Female body-mass index (kg/m^2^)^*^ | 22.3 (20.1, 24.5) | 22.0 (20.0, 24.6) | 0.591 | 0.555 |
| Duration of infertility (years)^*^ | 3 (2, 5) | 3 (2, 5) | 1.541 | 0.123 |
| Number of previous IVF/ICSI cycles |  |  |  |  |
| 0 | 435 (90.6%) | 429 (90.7%) | 0.001 | 0.969 |
| 1 | 45 (9.4%) | 44 (9.3%) |  |  |
| Primary infertility | 233 (48.5%) | 223 (47.1%) | 0.186 | 0.666 |
| Indication for IVF |  |  |  |  |
| Male factor (non-severe) | 480 (100%) | 473 (100%) | — | — |
| Female factor | 480 (100%) | 473 (100%) | — | — |
| Tubal factor | 344 (71.7%) | 349 (73.8%) | 0.539 | 0.463 |
| Ovulatory dysfunction | 48 (10.0%) | 47 (9.9%) | 0.001 | 0.974 |
| Endometriosis | 37 (7.7%) | 29 (6.1%) | 0.919 | 0.338 |
| Diminished ovarian reserve | 45 (9.4%) | 34 (7.2%) | 1.499 | 0.221 |
| Others | 38 (7.9%) | 41 (8.7%) | 0.177 | 0.674 |
| Ultrasonographic examination |  |  |  |  |
| Antral follicle count^*^ | 13 (8, 18) | 14 (9, 20) | 1.753 | 0.080 |
| Endometrial thickness (mm)^*^ | 7 (5, 9) | 6.9 (5, 9) | 0.006 | 0.995 |
| Basal laboratory testing (Female) |  |  |  |  |
| Basal follicle-stimulating hormone (IU/L)^*^ | 6.5 (5.2, 8.1) | 6.5 (5.2, 7.8) | 0.597 | 0.551 |
| Basal luteinizing hormone (IU/L)^*^ | 4.3 (2.9, 6.1) | 4.4 (3.0, 6.6) | 1.462 | 0.144 |
| Basal estradiol (pmol/L)^*^ | 130.6 (81.2, 195.0) | 140.0 (87.0, 194.9) | 0.587 | 0.557 |
| Basal semen analysis prior to IVF |  |  |  |  |
| Sperm volume (mL) | 3.0 (2.2, 4.1) | 3.0 (2.0, 4.0) | 1.235 | 0.217 |
| Sperm concentration (10^6^/mL) | 35.1 (17.4, 64.6) | 39.1 (20.3, 66.4) | 1.749 | 0.080 |
| Progressive motility (%) | 23.2 (17.2, 28.0) | 24.3 (18.7, 28.6) | 1.365 | 0.172 |
| Normal morphology (%)^*^ | 3.0 (2.0, 5.0) | 3.0 (2.0, 5.0) | 0.067 | 0.947 |
| DFI (%) | 18.8 (12.2, 26.3) | 18.4 (12.6, 25.1) | 0.069 | 0.945 |
| Classification of basal semen analysis |  |  |  |  |
| Oligoasthenozoospermia | 60 (12.5%) | 44 (9.3%) | 2.719 | 0.257 |
| Oligozoospermia | 38 (7.9%) | 35 (7.4%) |  |  |
| Asthenozoospermia | 382 (79.6%) | 394 (83.3%) |  |  |
| Controlled ovarian hyperstimulation protocol |  |  |  |  |
| GnRH-agonist protocol | 170 (35.4%) | 184 (38.9%) | 1.239 | 0.266 |
| GnRH-antagonist protocol | 310 (64.6%) | 289 (61.1%) |  |  |
| No. of days of ovarian stimulation | 10 (9, 12) | 10 (9, 12) | 0.150 | 0.881 |
| Total dose of follicle-stimulating hormone (IU) | 2025 (1500, 2700) | 2025 (1500, 2700) | 0.910 | 0.363 |
| hCG trigger day |  |  |  |  |
| Luteinizing hormone (IU/L)^*^ | 1.5 (0.8, 2.6) | 1.4 (0.8, 2.5) | 0.639 | 0.523 |
| Estradiol (pmol/L)^*^ | 9057.6 (4941.0, 13784.5) | 8446.5 (5389.0, 13820.0) | 0.301 | 0.764 |
| Progesterone (nmol/L)^*^ | 2.6 (1.6, 3.9) | 2.3 (1.6, 3.5) | 1.020 | 0.308 |
| Endometrial thickness (mm) | 10.4 (9.0, 12.0) | 11.0 (9.6, 12.6) | 2.515 | 0.012 |
| <8 mm | 26 (5.4%) | 26 (5.5%) | 3.582 | 0.167 |
| 8~12 mm | 350 (72.9%) | 320 (67.7%) |  |  |
| >12 mm | 104 (21.7%) | 127 (26.8%) |  |  |
| Progressive motile sperm for insemination on the day of oocyte retrieval (10^6^/mL) | 11.2 (6.4, 26.1) | 11.7 (7.1, 27.0) | 1.317 | 0.188 |
| No. of oocytes retrieved | 11 (7, 17) | 11 (7, 17) | 0.632 | 0.527 |
| No. of metaphase-II oocytes | 9 (5, 13) | — | — | — |

Data are n (%), n/N (%), or median (inter-quartile range). DFI=DNA fragmentation index.

^*^ The number of missing data was 0 in the ICSI group and 1 in the c-IVF group for female body-mass index; 1 and 0 for duration of infertility; 0 and 2 for antral follicle count, and 6 and 10 for endometrial thickness in ultrasonographic examination; 3 and 2 for basic follicle-stimulating hormone, basal luteinizing hormone, and basal estradiol in laboratory testing (female); 68 and 75 for normal morphology in basal semen analysis prior to IVF; 3 and 2 for luteinizing hormone, and 1 and 1 for estradiol, 0 and 2 for progesterone on the hCG trigger day.
