# Supplementary material for: Evaluating the potential for sperm DNA fragmentation testing to guide the use of ICSI for couples with non-severe male infertility
Source: Hum Reprod Open. 2026 Mar 7;2026(2):hoag011. doi: 10.1093/hropen/hoag011 (PMC12981913; doi:10.1093/hropen/hoag011)
Supplement: hoag011_Supplementary_Data [file hoag011_supplementary_data.zip › Supplementary-Table-S4-post adjudication clean.docx]

**Supplementary Table S4: Treatment effects on embryological outcomes in each** **quartile of sperm DFI**

|  | **ICSI** | **IVF** | **Adjusted OR (95% CI)** | **Adjusted p value** |
| --- | --- | --- | --- | --- |
| Number of available embryos on day 3 |  |  |  |  |
| Q1 (DFI<12.3%) | 5 (2, 8) | 5 (2.5, 9) | — | 0.095 |
| Q2 (12.3≤DFI<18.6%) | 6 (3, 10) | 6 (3, 10) | — | 0.744 |
| Q3 (18.6≤DFI<25.9%) | 5 (2, 8) | 5 (3, 9) | — | 0.365 |
| Q4 (DFI≥25.9%) | 5 (2, 8.75) | 5 (2, 10) | — | 0.613 |
| Number of good quality embryos on day 3 |  |  |  |  |
| Q1 (DFI<12.3%) | 3 (1, 6) | 3 (1, 6) | — | 0.441 |
| Q2 (12.3≤DFI<18.6%) | 3 (1, 6) | 3 (1, 6) | — | 0.901 |
| Q3 (18.6≤DFI<25.9%) | 2 (1, 5) | 3 (1, 6) | — | 0.623 |
| Q4 (DFI≥25.9%) | 3 (1, 6) | 3 (1, 6) | — | 0.497 |
| Fresh embryo transfer in the first cycle |  |  |  |  |
| Q1 (DFI<12.3%) | 51/120 (42.5%) | 68/117 (58.1%) | 0.57 (0.33, 0.98) | 0.042 |
| Q2 (12.3≤DFI<18.6%) | 61/114 (53.5%) | 73/123 (59.3%) | 0.81 (0.48, 1.40) | 0.464 |
| Q3 (18.6≤DFI<25.9%) | 72/122 (59.0%) | 70/119 (58.8%) | 0.81 (0.47, 1.42) | 0.469 |
| Q4 (DFI≥25.9%) | 59/124 (47.6%) | 56/114 (49.1%) | 1.07 (0.62, 1.84) | 0.819 |
| Frozen-thawed embryo transfer in the first cycle |  |  |  |  |
| Q1 (DFI<12.3%) | 53/120 (44.2%) | 42/117 (35.9%) | 1.28 (0.73, 2.26) | 0.390 |
| Q2 (12.3≤DFI<18.6%) | 49/114 (43.0%) | 42/123 (34.1%) | 1.45 (0.84, 2.50) | 0.187 |
| Q3 (18.6≤DFI<25.9%) | 42/122 (34.4%) | 44/119 (37.0%) | 1.12 (0.62, 2.00) | 0.711 |
| Q4 (DFI≥25.9%) | 58/124 (46.8%) | 43/114 (37.7%) | 1.35 (0.76, 2.40) | 0.307 |
| Cleavage-stage embryo transfer in the first cycle |  |  |  |  |
| Q1 (DFI<12.3%) | 82/120 (68.3%) | 94/117 (80.3%) | 0.43 (0.21, 0.87) | 0.020 |
| Q2 (12.3≤DFI<18.6%) | 81/114 (71.1%) | 88/123 (71.5%) | 1.11 (0.56, 2.20) | 0.768 |
| Q3 (18.6≤DFI<25.9%) | 77/122 (63.1%) | 70/119 (58.8%) | 0.87 (0.45, 1.68) | 0.674 |
| Q4 (DFI≥25.9%) | 75/124 (60.5%) | 62/114 (54.4%) | 1.67 (0.91, 3.08) | 0.100 |
| Blastocyst embryo transfer in the first cycle |  |  |  |  |
| Q1 (DFI<12.3%) | 22/120 (18.3%) | 16/117 (13.7%) | 1.61 (0.68, 3.77) | 0.276 |
| Q2 (12.3≤DFI<18.6%) | 29/114 (25.4%) | 27/123 (22.0%) | 1.21 (0.56, 2.61) | 0.634 |
| Q3 (18.6≤DFI<25.9%) | 38/122 (31.1%) | 44/119 (37.0%) | 1.07 (0.52, 2.18) | 0.855 |
| Q4 (DFI≥25.9%) | 43/124 (34.7%) | 37/114 (32.5%) | 0.93 (0.46, 1.86) | 0.838 |

DFI=DNA fragmentation index. OR=Odds ratio.

Adjusted ORs (95% CIs) and p values were calculated by using logistic/linear model with the adjustment of centre.
