# Supplementary material for: Evaluating the potential for sperm DNA fragmentation testing to guide the use of ICSI for couples with non-severe male infertility
Source: Hum Reprod Open. 2026 Mar 7;2026(2):hoag011. doi: 10.1093/hropen/hoag011 (PMC12981913; doi:10.1093/hropen/hoag011)
Supplement: hoag011_Supplementary_Data [file hoag011_supplementary_data.zip › Supplementary-Table-S1-post adjudication clean.docx]

**Supplementary Table S1: Baseline characteristics of the study population included and excluded in the ICSI group**

| **Characteristic** | **Included couples**  **(N=480)** | **Excluded couples**  **(N=666)** | **Z/ꭓ^2^** | **P** |
| --- | --- | --- | --- | --- |
| Centre |  |  |  |  |
| 1 | 138 (28.7%) | 282 (42.3%) | 392.190 | <0.001 |
| 2 | 53 (11.0%) | 27 (4.1%) |  |  |
| 3 | 35 (7.3%) | 84 (12.6%) |  |  |
| 4 | 54 (11.3%) | 49 (7.4%) |  |  |
| 5 | 72 (15.0%) | 4 (0.6%) |  |  |
| 6 | 0 (0.0%) | 25 (3.8%) |  |  |
| 7 | 52 (10.8%) | 21 (3.2%) |  |  |
| 8 | 0 (0.0%) | 75 (11.3%) |  |  |
| 9 | 76 (15.8%) | 4 (0.6%) |  |  |
| 10 | 0 (0.0%) | 95 (14.3%) |  |  |
| Age (years) |  |  |  |  |
| Female | 34 (31, 38) | 34 (31, 37) | 1.110 | 0.267 |
| Male | 35 (31, 39) | 34 (31, 38) | 2.660 | 0.008 |
| Female body-mass index (kg/m^2^)^*^ | 22.3 (20.1, 24.5) | 22.0 (20.1, 24.8) | 0.216 | 0.829 |
| Duration of infertility (years)^*^ | 3 (2, 5) | 3 (2, 4) | 0.716 | 0.474 |
| Number of previous IVF/ICSI cycles |  |  |  |  |
| 0 | 435 (90.6%) | 607 (91.1%) | 0.090 | 0.764 |
| 1 | 45 (9.4%) | 59 (8.9%) |  |  |
| Primary infertility | 233 (48.5%) | 376 (56.5%) | 7.018 | 0.008 |
| Indication for IVF |  |  |  |  |
| Male factor (non-severe) | 480 (100%) | 666 (100%) | — | — |
| Female factor | 480 (100%) | 666 (100%) | — | — |
| Tubal factor | 344 (71.7%) | 390 (58.6%) | 20.815 | <0.001 |
| Ovulatory dysfunction | 48 (10.0%) | 82 (12.3%) | 1.483 | 0.223 |
| Endometriosis | 37 (7.7%) | 53 (8.0%) | 0.024 | 0.877 |
| Diminished ovarian reserve | 45 (9.4%) | 68 (10.2%) | 0.219 | 0.640 |
| Others | 38 (7.9%) | 30 (4.5%) | 5.819 | 0.016 |
| Ultrasonographic examination |  |  |  |  |
| Antral follicle count^*^ | 13 (8, 18) | 12 (8, 17) | 2.078 | 0.038 |
| Endometrial thickness (mm)^*^ | 7 (5, 9) | 7 (5, 9) | 0.911 | 0.362 |
| Basal laboratory testing (Female) |  |  |  |  |
| Basal follicle-stimulating hormone (IU/L)^*^ | 6.5 (5.2, 8.1) | 6.7 (5.5, 8.1) | 1.246 | 0.213 |
| Basal luteinizing hormone (IU/L)^*^ | 4.3 (2.9, 6.1) | 4.1 (3.0, 5.6) | 1.069 | 0.285 |
| Basal estradiol (pmol/L)^*^ | 130.6 (81.2, 195.0) | 145.0 (100.8, 198.1) | 2.253 | 0.024 |
| Basal semen analysis prior to IVF |  |  |  |  |
| Sperm volume (mL) | 3.0 (2.2, 4.1) | 2.7 (2.0, 3.6) | 4.214 | <0.001 |
| Sperm concentration (10^6^/mL) | 35.1 (17.4, 64.6) | 39.8 (15.9, 70.4) | 1.303 | 0.193 |
| Progressive motility (%) | 23.2 (17.2, 28.0) | 22.0 (16.5, 27.5) | 2.103 | 0.035 |
| Normal morphology (%)^*^ | 3.0 (2.0, 5.0) | 3.0 (2.0, 4.0) | 1.162 | 0.245 |
| DFI (%) | 18.8 (12.2, 26.3) | — | — | — |
| Classification of basal semen analysis |  |  |  |  |
| Oligoasthenozoospermia | 60 (12.5%) | 102 (15.3%) | 1.978 | 0.372 |
| Oligozoospermia | 38 (7.9%) | 47 (7.1%) |  |  |
| Asthenozoospermia | 382 (79.6%) | 517 (77.6%) |  |  |
| Controlled ovarian hyperstimulation protocol |  |  |  |  |
| GnRH-agonist protocol | 170 (35.4%) | 277 (41.6%) | 4.471 | 0.034 |
| GnRH-antagonist protocol | 310 (64.6%) | 389 (58.4%) |  |  |
| No. of days of ovarian stimulation | 10 (9, 12) | 10 (9, 12) | 0.738 | 0.461 |
| Total dose of follicle-stimulating hormone (IU) | 2025 (1500, 2700) | 2025 (1500, 2700) | 0.357 | 0.721 |
| hCG trigger day |  |  |  |  |
| Luteinizing hormone (IU/L)^*^ | 1.5 (0.8, 2.6) | 1.5 (0.8, 2.8) | 1.413 | 0.158 |
| Estradiol (pmol/L)^*^ | 9057.6 (4941.0, 13784.5) | 8507.3 (5029.2, 13851.7) | 0.535 | 0.593 |
| Progesterone (nmol/L)^*^ | 2.6 (1.6, 3.9) | 2.7 (1.7, 5.0) | 3.490 | <0.001 |
| Endometrial thickness (mm) | 10.4 (9.0, 12.0) | 11.0 (10, 12) | 2.956 | 0.003 |
| <8 mm | 26 (5.4%) | 30 (4.5%) | 0.970 | 0.616 |
| 8~12 mm | 350 (72.9%) | 478 (71.8%) |  |  |
| >12 mm | 104 (21.7%) | 157 (23.6%) |  |  |
| Progressive motile sperm for insemination on the day of oocyte retrieval (10^6^/mL) | 11.2 (6.4, 26.1) | 6.5 (2.4, 12.5) | 9.971 | <0.001 |
| No. of oocytes retrieved | 11 (7, 17) | 10 (7, 16) | 1.951 | 0.051 |
| No. of metaphase-II oocytes | 9 (5, 13) | 8 (5, 12) | 2.481 | 0.013 |

Data are n (%), n/N (%), or median (inter-quartile range). IVF=conventional in vitro fertilisation. DFI=DNA fragmentation index.

* The number of missing data was 1 in the included group and 0 in the excluded group for duration of infertility; 0 and 1 for antral follicle count, and 6 and 11 for endometrial thickness in ultrasonographic examination; 3 and 8 for basic follicle-stimulating hormone, 3 and 6 for basal luteinizing hormone, 3 and 5 for basal estradiol in laboratory testing (female); 68 and 105 for normal morphology in basal semen analysis prior to IVF; 3 and 6 for luteinizing hormone, 1 and 4 for estradiol, 0 and 13 for progesterone, and 0 and 1 for endometrial thickness on the hCG trigger day.
