# Supplementary material for: Evaluating the potential for sperm DNA fragmentation testing to guide the use of ICSI for couples with non-severe male infertility
Source: Hum Reprod Open. 2026 Mar 7;2026(2):hoag011. doi: 10.1093/hropen/hoag011 (PMC12981913; doi:10.1093/hropen/hoag011)
Supplement: hoag011_Supplementary_Data [file hoag011_supplementary_data.zip › Supplementary-Table-S2-post adjudication clean.docx]

**Supplementary Table S2: Baseline characteristics of the study population included and excluded in the IVF group**

| **Characteristic** | **Included couples**  **(N=473)** | **Excluded couples**  **(N=710)** | **Z/ꭓ^2^** | **P** |
| --- | --- | --- | --- | --- |
| Centre |  |  |  |  |
| 1 | 128 (27.1%) | 294 (41.4%) | 422.673 | <0.001 |
| 2 | 45 (9.5%) | 32 (4.5%) |  |  |
| 3 | 40 (8.5%) | 113 (15.9%) |  |  |
| 4 | 62 (13.1%) | 27 (3.8%) |  |  |
| 5 | 68 (14.4%) | 6 (0.8%) |  |  |
| 6 | 0 (0.0%) | 25 (3.5%) |  |  |
| 7 | 49 (10.4%) | 28 (3.9%) |  |  |
| 8 | 0 (0.0%) | 75 (10.6) |  |  |
| 9 | 81 (17.1%) | 3 (0.4%) |  |  |
| 10 | 0 (0.0%) | 107 (15.1%) |  |  |
| Age (years) |  |  |  |  |
| Female | 34 (30, 38) | 33 (31, 36) | 0.867 | 0.386 |
| Male | 34 (31, 39) | 34 (31, 38) | 1.903 | 0.057 |
| Female body-mass index (kg/m^2^)^*^ | 22.0 (20.0, 24.6) | 22.2 (20.3, 24.8) | 1.361 | 0.174 |
| Duration of infertility (years)^*^ | 3 (2, 5) | 3 (2, 5) | 2.004 | 0.045 |
| Number of previous IVF/ICSI cycles |  |  |  |  |
| 0 | 429 (90.7%) | 648 (91.3%) | 0.113 | 0.737 |
| 1 | 44 (9.3%) | 62 (8.7%) |  |  |
| Primary infertility | 223 (47.1%) | 388 (54.6%) | 6.398 | 0.011 |
| Indication for IVF |  |  |  |  |
| Male factor (non-severe) | 473 (100%) | 710 (100%) | — | — |
| Female factor | 473 (100%) | 710 (100%) | — | — |
| Tubal factor | 349 (73.8%) | 438 (61.7%) | 18.646 | <0.001 |
| Ovulatory dysfunction | 47 (9.9%) | 106 (14.9%) | 6.285 | 0.012 |
| Endometriosis | 29 (6.1%) | 52 (7.3%) | 0.633 | 0.426 |
| Diminished ovarian reserve | 34 (7.2%) | 72 (10.1%) | 3.034 | 0.082 |
| Others | 41 (8.7%) | 42 (5.9%) | 3.297 | 0.069 |
| Ultrasonographic examination |  |  |  |  |
| Antral follicle count^*^ | 14 (9, 20) | 13 (8, 19) | 2.386 | 0.017 |
| Endometrial thickness (mm)^*^ | 6.9 (5, 9) | 6 (5, 9) | 1.239 | 0.215 |
| Basal laboratory testing (Female) |  |  |  |  |
| Basal follicle-stimulating hormone (IU/L)^*^ | 6.5 (5.2, 7.8) | 6.6 (5.3, 8.0) | 0.639 | 0.523 |
| Basal luteinizing hormone (IU/L)^*^ | 4.4 (3.0, 6.6) | 4.0 (2.7, 6.0) | 2.601 | 0.009 |
| Basal estradiol (pmol/L)^*^ | 140.0 (87.0, 194.9) | 146.8 (97.5, 195.8) | 1.239 | 0.215 |
| Basal semen analysis prior to IVF |  |  |  |  |
| Sperm volume (mL) | 3.0 (2.0, 4.0) | 3.0 (2.0, 3.7) | 2.036 | 0.042 |
| Sperm concentration (10^6^/mL) | 39.1 (20.3, 66.4) | 42.9 (21.0, 70.0) | 1.207 | 0.227 |
| Progressive motility (%) | 24.3 (18.7, 28.6) | 22.8 (17.2, 28.0) | 2.717 | 0.007 |
| Normal morphology (%)^*^ | 3.0 (2.0, 5.0) | 3.0 (2.0, 4.0) | 1.732 | 0.083 |
| DFI (%) | 18.4 (12.6, 25.1) | — | — | — |
| Classification of basal semen analysis |  |  |  |  |
| Oligoasthenozoospermia | 44 (9.3%) | 73 (10.3%) | 0.381 | 0.827 |
| Oligozoospermia | 35 (7.4%) | 55 (7.7%) |  |  |
| Asthenozoospermia | 394 (83.3%) | 582 (82.0%) |  |  |
| Controlled ovarian hyperstimulation protocol |  |  |  |  |
| GnRH-agonist protocol | 184 (38.9%) | 295 (41.5%) | 0.827 | 0.363 |
| GnRH-antagonist protocol | 289 (61.1%) | 415 (58.5%) |  |  |
| No. of days of ovarian stimulation | 10 (9, 12) | 10 (9, 12) | 0.616 | 0.538 |
| Total dose of follicle-stimulating hormone (IU) | 2025 (1500, 2700) | 2000 (1500, 2700) | 0.203 | 0.839 |
| hCG trigger day |  |  |  |  |
| Luteinizing hormone (IU/L)^*^ | 1.4 (0.8, 2.5) | 1.6 (0.8, 3.1) | 2.147 | 0.032 |
| Estradiol (pmol/L)^*^ | 8446.5 (5389.0, 13820.0) | 8657.0 (5238.0, 14486.7) | 0.146 | 0.884 |
| Progesterone (nmol/L)^*^ | 2.3 (1.6, 3.5) | 2.8 (1.7, 5.1) | 5.170 | <0.001 |
| Endometrial thickness (mm) | 11.0 (9.6, 12.6) | 11.0 (9.7, 12.0) | 1.115 | 0.265 |
| <8 mm | 26 (5.5%) | 40 (5.6%) | 6.077 | 0.048 |
| 8~12 mm | 320 (67.7%) | 523 (73.7%) |  |  |
| >12 mm | 127 (26.8%) | 147 (20.7%) |  |  |
| Progressive motile sperm for insemination on the day of oocyte retrieval (10^6^/mL) | 11.7 (7.1, 27.0) | 8.0 (4.7, 18.6) | 7.402 | <0.001 |
| No. of oocytes retrieved | 11 (7, 17) | 11 (7, 17) | 0.612 | 0.541 |
| No. of metaphase-II oocytes | — | — | — | — |

Data are n (%), n/N (%), or median (inter-quartile range). DFI=DNA fragmentation index.

^*^ The number of missing data was 1 in the included group and 3 in the excluded group for female body-mass index; 2 and 0 for antral follicle count, and 10 and 4 for endometrial thickness in ultrasonographic examination; 2 and 7 for basic follicle-stimulating hormone, 2 and 7 for basal luteinizing hormone, and 2 and 9 basal estradiol in laboratory testing (female); 75 and 116 for normal morphology in basal semen analysis prior to IVF; 2 and 2 for luteinizing hormone, and 1 and 2 for estradiol, 2 and 11 for progesterone on the hCG trigger day.
